# Apigenin: Selective CK2 inhibitor increases Ikaros expression and improves T cell homeostasis and function in murine pancreatic cancer

**DOI:** 10.1371/journal.pone.0170197

**Published:** 2017-02-02

**Authors:** Nadine Nelson, Karoly Szekeres, Cristina Iclozan, Ivannie Ortiz Rivera, Andrew McGill, Gbemisola Johnson, Onyekachi Nwogu, Tomar Ghansah

**Affiliations:** 1 Department of Molecular Medicine, University of South Florida, Tampa, Florida, United States of America; 2 Department of Immunology, Moffitt Cancer Center, Tampa, Florida, United States of America; Pennsylvania State University, UNITED STATES

## Abstract

Pancreatic cancer (PC) evades immune destruction by favoring the development of regulatory T cells (Tregs) that inhibit effector T cells. The transcription factor Ikaros is critical for lymphocyte development, especially T cells. We have previously shown that downregulation of Ikaros occurs as a result of its protein degradation by the ubiquitin-proteasome system in our Panc02 tumor-bearing (TB) mouse model. Mechanistically, we observed a deregulation in the balance between Casein Kinase II (CK2) and protein phosphatase 1 (PP1), which suggested that increased CK2 activity is responsible for regulating Ikaros’ stability in our model. We also showed that this loss of Ikaros expression is associated with a significant decrease in CD4^+^ and CD8^+^ T cell percentages but increased CD4^+^CD25^+^ Tregs in TB mice. In this study, we evaluated the effects of the dietary flavonoid apigenin (API), on Ikaros expression and T cell immune responses. Treatment of splenocytes from naïve mice with (API) stabilized Ikaros expression and prevented Ikaros downregulation in the presence of murine Panc02 cells *in vitro*, similar to the proteasome inhibitor MG132. *In vivo* treatment of TB mice with apigenin (TB-API) improved survival, reduced tumor weights and prevented splenomegaly. API treatment also restored protein expression of some Ikaros isoforms, which may be attributed to its moderate inhibition of CK2 activity from splenocytes of TB-API mice. This partial restoration of Ikaros expression was accompanied by a significant increase in CD4^+^ and CD8^+^ T cell percentages and a reduction in Treg percentages in TB-API mice. In addition, CD8^+^ T cells from TB-API mice produced more IFN-γ and their splenocytes were better able to prime allogeneic CD8^+^ T cell responses compared to TB mice. These results provide further evidence that Ikaros is regulated by CK2 in our pancreatic cancer model. More importantly, our findings suggest that API may be a possible therapeutic agent for stabilizing Ikaros expression and function to maintain T cell homeostasis in murine PC.

## Introduction

Pancreatic cancer (PC) is one of the most aggressive and most lethal solid malignancies [1]. The pancreatic tumor microenvironment favors the recruitment of immunosuppressive cells that dampen anti-tumor immune responses, allowing tumor cells to evade immune surveillance and leading to tumor progression [2, 3]. Understanding the mechanisms by which these anti-tumor immune responses, specifically those mediated by T cells, are regulated in PC is therefore critical for developing new, targeted treatment options.

Effector CD4^+^ and CD8^+^ T cells play important roles in the host’s immune response to cancer [4]. Early studies showed a conventional “helper” role for CD4^+^ T cells by primarily influencing immune responses by regulating CD8^+^ cytotoxic T lymphocytes (CTLs) [5]. The percentages and function of CD8^+^ T cells are significantly decreased in the peripheral blood of PC patients, compared to healthy controls [6]. One contributing mechanism to this diminished anti-tumor response in PC patients is the induction and recruitment of suppressive cells by tumor-derived factors (TDF) [2, 3]. In particular, immunosuppressive regulatory T cells (Tregs) are a subpopulation of CD4^+^ T cells that express the forkhead boxP3 (FoxP3) gene [7]. Their main function is maintaining peripheral immune tolerance against self-antigens and foreign antigens by suppressing CD4^+^ and CD8^+^ T cell responses [8]. The percentages of Tregs are elevated in PC in human patients as well as murine models of PC [9–11]. Delineating the mechanisms by which this balance in T cells is lost is critical for the generation of effective anti-tumor immune responses in PC hosts.

Alterations in transcription factors (TF) that play critical roles in the commitment and maintenance of lymphocyte development often promote malignant transformation [12]. One such example is the Ikaros family of zinc finger TF that includes Ikaros, Aiolos, Helios, Eos and Pegasus proteins. Ikaros, Helios and Aiolos are restricted to the immune-cell lineages whereas Eos and Pegasus are found in lymphoid tissues [13]. These TF regulate cell-fate decisions during hematopoiesis and are thus important players in the development of immune cells [13]. In particular, Ikaros, the founding member is highly important for normal T cell development [14–16]. Ikaros is regulated post-transcriptionally by alternative splicing, which produces functional and dominant-negative (DN) isoforms, which can inhibit its activity [17, 18]. Ikaros is also regulated by posttranslational modifications, which primarily include phosphorylation [19]. Phosphorylation by protein kinase (Casein II) CK2 and dephosphorylation by protein phosphatase 1 (PP1) can negatively affect Ikaros’ stability, localization and function [20]. Specifically, CK2 phosphorylation of Ikaros impairs its DNA binding ability, regulation of cell cycle progression, and its function in T cells. It also alters its subcellular localization and leads to its ubiquitin-mediated proteasomal degradation via phosphorylation in PEST sequence regions [20–22]. On the contrary, dephosphorylation of Ikaros by PP1 maintains its stability and function [20, 21, 23]. CK2 is a ubiquitously expressed and highly conserved serine/threonine kinase that regulates a number of critical cellular processes, including cell proliferation and apoptosis [24–26]. CK2 is widely studied in blood and solid malignancies [27]. Overexpression of its tetrameric subunits and deregulation of its activity have been linked to numerous cancers [24]. Overexpression of CK2 in mice leads to T cell leukemia’s and lymphomas [28–30]. However, limited studies have focused on CK2’s involvement in regulating immune responses.

Apigenin (API) is a natural plant flavonoid and selective CK2 inhibitor that targets CK2-dependent signaling pathways. API has a number of reported biological effects including anti-proliferative, anti-oxidant, anti-inflammatory and anti-carcinogenic characteristics, which are thought to be an integral part of its anti-cancer attributes [31]. Recently, there has been increased exploration of the use of API as a chemopreventive agent in a number of cancer models [32]. More specifically, API has been shown to induce cell death and also enhance the anti-proliferative effects of chemotherapeutic agents in human PC cells, *in vitro* [33–35].

We have previously shown that Ikaros undergoes proteasomal degradation, which may contribute to altered effector and regulatory T cell development in murine PC [36]. Our studies suggest that a shift in the balance between CK2 and PP1, favoring CK2 activity may be responsible. Therefore, to further delineate CK2’s involvement in regulating Ikaros expression and thus T cell responses, we investigated the effects of API in our PC model. We found that API is able to stabilize Ikaros’ expression *in vitro* and *in vivo* while also restoring the balance between effector CD4^+^/CD8^+^ T cells and Tregs. This correlated with an increase in immune function as observed on splenocytes from API treated pancreatic tumor-bearing (TB-API) mice exemplified by increase in the *in vivo* production of INF-γ CD8^+^ T cells *in vivo* and by robust allogeneic CD8^+^ T cell responses, *in vitro*. This study highlights the importance of CK2 in regulating Ikaros expression and its possible influence on T cell immune responses in murine PC.

## Materials and methods

### Cell line

Panc02 murine pancreatic adenocarcinoma cell line was established by Corbett et al. [37]. This cell line was maintained in complete RPMI 1640 medium supplemented with 10% Fetal Bovine Serum (FBS), (HyClone, Logan, UT), 2mM L-glutamine, 100μ/ml penicillin and 100μg/ml streptomycin (Gibco BRL, Rockville, MD) at 37°C in 5% CO_2_. Cultured cells were tested and found to be negative for mycoplasma and viral contamination.

### Mice

Female C57BL/6N mice (6–8 weeks) were purchased from Harlan Laboratories (Indianapolis). The Institutional Animal Care and Use Committee of the University of South Florida approved protocol T IS00000447 is in compliance with the Guide for the Care and Use of Laboratory Animals. All mice were maintained in a pathogen-free animal facility, fed and housed with other mice for 1 week before the start of *in vitro* or *in vivo* experiments. Mice were humanely euthanized using CO_2_ and cervical dislocation according to the University of South Florida IACUC guidelines.

### CK2 inhibitor

Apigenin (CK2 Inhibitor) (API) **(**4′,5,7-Trihydroxyflavone, 5,7-Dihydroxy-2-(4-hydroxyphenyl)-4-benzopyrone**)** was purchased from Fisher Scientific, USA and diluted in DMSO according to the manufacturer’s instructions.

### Proteasome inhibitor

MG132 (proteasome inhibitor Cbz-LLL) carbobenzoxyl-L-leucyl-leucyl-L-leucine was purchased from Fisher Scientific, USA and diluted in DMSO according to manufacturer’s instructions.

### In vitro assay

Control splenocytes from C57BL/6N mice were collected and co-cultured in the absence or presence of Panc02 cells, treated with and without API or MG132 for four hours at 10μM and 20μM, *in vitro*. Cells were harvested for protein lysates and western blot analysis.

### Mice

Female C57BL/6N mice (6–8 weeks of age) were injected with 1.5 × 10^5^ Panc02 cells suspended in 100μl of PBS and administered subcutaneously (s.c.) in the lower, left abdomen. Mice from the control (CTRL) group were s.c. injected with sterile PBS only. Treatments of pancreatic tumor-bearing (TB) mice started immediately after the tumor onset (as evidenced by the appearance of palpable tumors). A group of TB mice received either 100μl of PBS (TB) or doses of 25mg/kg of API (TB-API) administered three times per week via intra-peritoneal injections (i.p.). Mice were monitored three times per week for weight, infection, abdominal swelling (due to ascites), impediment in locomotion, labored breathing, and any signs of discomfort (pain). At the experimental endpoint of TB and TB-API mice, from the survival or treatment studies that experienced, signs of suffering or pain, abdominal swelling due to ascites (the main cause of animal deaths), solitary tumor masses greater than 2 cm or necrotic tumors were humanely euthanized using CO_2_ and cervical dislocation, according to the University of South Florida IACUC guidelines and approved protocol (T IS0000447). Tumors and spleens were harvested and weighed from all mice. In addition, spleens were processed for *in vivo* and *in vitro* biochemical experiments.

### In vitro CK2 kinase assay

CK2 kinase activity was measured in splenocytes from CTRL, TB and TB-API mice using the CK2 assay kit (Millipore) according to the manufacturer’s instructions [36]. CK2 activity was calculated by subtracting the mean counts per minute (CPM) of samples in the absence of substrate from the mean CPM of samples in the presence of substrate [36].

### Western blot analyses

Protein lysates were prepared from splenocytes from CTRL, TB and TB-API mice. In addition, control splenocytes were co-incubated in the absence or presence of Panc02 cells treated with and without API and/or MG132. Cells were lysed with modified Radioimmunoprecipitation assay (RIPA) Buffer (Millipore) supplemented with Na_3_OV_4_ and protease inhibitor cocktail (Sigma-Aldrich). Protein concentrations were determined using the BCA Protein Assay Kit (Thermo Fisher Scientific). Approximately, 40 μg protein lysates were loaded and resolved using NuPAGE 4–12% Bis-Tris polyacrylamide gels (Invitrogen) and transferred to nitrocellulose membranes (Whatman). The membranes were blocked with 5% nonfat milk in PBS/0.1% Tween-20 and then probed with either anti-Ikaros (Cell Signaling), at a dilution of 1:1000, anti-CK2α (Santa Cruz Biotechnology) and anti-PP1 (Santa Cruz Biotechnology) at a dilution of 1:200. Primary antibodies were detected using their respective secondary IgG, HRP-conjugated antibodies (Jackson Immunoresearch), at a dilution of 1:10000. Secondary antibodies were identified using Super Signal West Pico and Femto Chemiluminescent Substrates (Thermo Fisher Scientific). As an internal control for equal protein loading, all blots were stripped and re-probed with anti-β-actin (Sigma-Aldrich) at a dilution of 1:20,000 or anti-GAPDH (Santa Cruz Biotechnology) at a dilution of 1:200. Membranes were either exposed to X-ray films (Phoenix) and developed using a Kodak M35-X OMAT Processor or imaged using a ChemiDoc XRS Imaging System (Bio-Rad). Band intensities were quantified using Quantity One 1-D Densitometry and Image Lab softwares (Bio-Rad) [36].

### Flow cytometry

Splenocytes from CTRL, TB and TB-API mice were lysed with red blood cell (RBC) lysis buffer (eBioscience) and counted for immunophenotyping. Cells were then suspended in 3% FBS-PBS and stained with fluorescent antibodies against murine T cell surface markers CD3 (FITC) (eBioscience), CD4 (PE-Cy7) (BD Pharmingen), CD8 (APC-H7) (BD Pharmingen) and CD25 (PE) (eBioscience). Subsequently, cells were intracellularly stained with anti-IFN-γ-PE (BD Pharmigen) after using a fixation-permeabilization kit from eBioscience according to the manufacturers protocol. Flow cytometry was performed using a BD LSRII (BD Biosciences Immunocytometry Systems) and data analyzed with FlowJo software (Tree Star Inc.) [36].

### Allogeneic mixed lymphocyte reaction

CTRL, TB and TB-API spleens and Balb/c spleens were processed into single cell suspensions, RBC lysed and counted. 4 × 10^5^/well Balb/c splenocytes (responders) were labeled with 1μM of Carboxyfluorescein diacetate succinimidyl ester (CFSE) and co-cultured with 8 × 10^5^/well irradiated (2000 rad) C57BL/6N splenocytes (stimulators) from CTRL, TB and TB-API mice. Culture wells were set-up in triplicate in a 96 well plate in a one-way allogeneic mixed-leukocyte reaction (MLR), and cultured for 4 days at 37°C [38]. Proliferation responses of allogeneic CD8^+^ T cells from Balb/c mice assay were evaluated using flow cytometery. Cells were stained with murine anti-CD3 PerCP (BD Pharmigen), anti-CD8-APC-H7 (BD Pharmigen). The CFSE dilution profile of CFSE^+^CD3^+^CD8^+^ cells was acquired using BD LSRII flow cytometer and data analyzed with FlowJo software (Tree Star Inc.).

### Statistical analysis

All *in vivo* and *in vitro* graph results described in this study are representative of the mean ± S.E.M. of at least three independent experiments analyzed with two-tailed Student’s *t* test and Kaplan–Meier survival curve using Prism 5 Software (GraphPad, San Diego, CA). Statistical significance and representative quantification of normalized densitometric ratios of western blot data was realized using the *J* software. Statistical differences were considered significant at p< 0.05.

## Results

### Apigenin prevents Ikaros downregulation, in vitro

**Fig 1:**
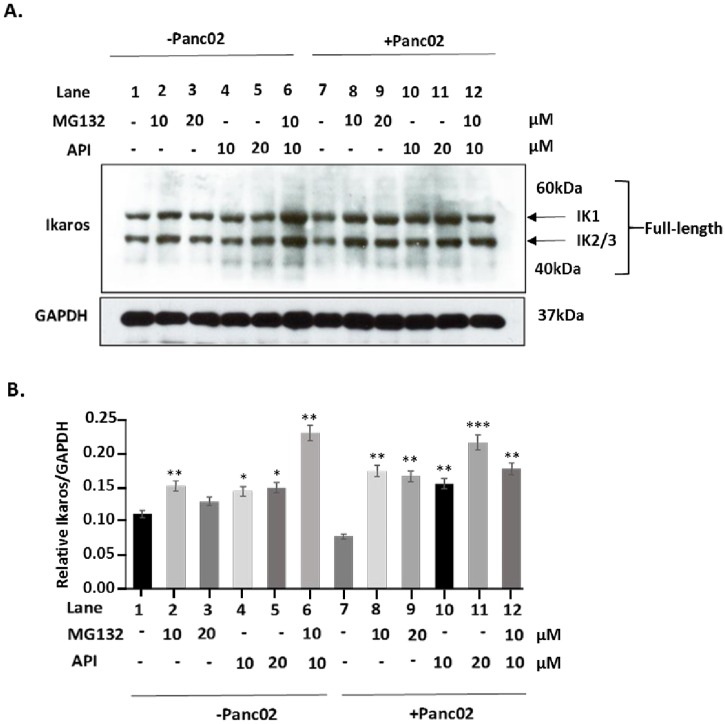
Apigenin prevents Ikaros downregulation, in vitro. **(**A) Western blot analysis of Ikaros in naïve splenocytes co-cultured in the absence or presence of Panc02 cells, treated with apigenin (API) and/or MG132 for four hours at 10μM and 20μM, *in vitro*. To control for equal protein loading the blot was reprobed with an antibody specific to GAPDH. (B**)** Representative quantification of normalized densitometric ratios of western blot data is shown. Graph represented is the mean ± S.E.M. of three independent experiments. Lanes 1–6 vs. Lane 1; Lane 7 vs. Lane 1; Lanes 8–12 vs. Lane 7 *p<0.05, **p<0.005; ***p<0.0001(by two-tailed Student’s *t* test).

We previously published that MG132 is able to stabilize Ikaros expression *in vitro*, providing evidence that Ikaros undergoes ubiquitin proteasomal degradation [36]. A balance between CK2 and PP1 regulates Ikaros stability and function [20, 21, 39]. In particular, increased CK2 activity is thought to cause Ikaros degradation [21]. Therefore, inhibiting CK2 should stabilize Ikaros expression and prevent its degradation, similar to MG132. We treated naïve splenocytes with the CK2 inhibitor, API, as well as MG132, both at 10μM and 20μM, to compare their effects on Ikaros expression. Both API and MG132 stabilized Ikaros expression (Fig 1A and 1B Lanes 2 and 3 vs. Lane 1; Lanes 4 and 5 vs. Lane 1), respectively and displayed a significant synergistic effect, which shows accumulation of ubiquitination ladders (Fig 1A and 1B Lane 6). The addition of murine Panc02 cells causes a reduction, although not significant, in Ikaros protein expression (Fig 1A and 1B Lane 7) that is prevented by MG132 treatment. To determine whether API has the same activity as MG132 to prevent downregulation of Ikaros, we treated Panc02 cells and naïve splenocyte co-cultures with both 10μM and 20μM of API (Fig 1A Lanes 10 and 11) and 10μM and 20μM of MG132 (Fig 1A Lanes 8 and 9). Thus, our results show that API treatment also significantly prevented Panc02 reduction of Ikaros protein expression in splenocytes at the same concentration as MG132 (Fig 1B). However, adding both drugs did not result in a synergistic effect in this co-culture system (Fig 1A Lane 12). Overall, these results suggest that API is able to stabilize Ikaros and prevent its downregulation in a pancreatic tumor microenvironment. Moreover, the similarities to MG132 and their additive effect, also further suggest that API may be preventing Ikaros’ proteasomal degradation, possibly via its inhibition of CK2.

### Apigenin increases survival and reduces tumor burden, in vivo

**Fig 2:**
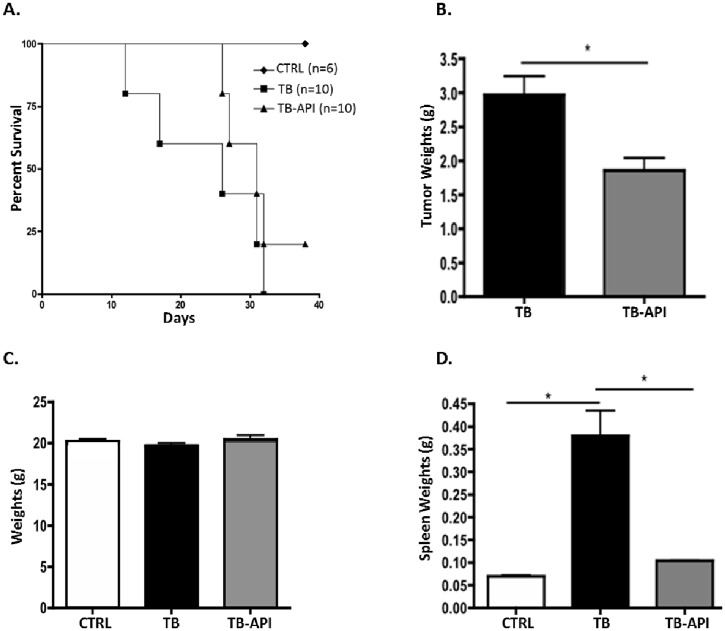
Apigenin increases survival and reduces tumor burden in TB mice, in vivo. **(**A) Kaplan-Meier survival curve show that Control (CTRL) (n = 6), TB-API mice (n = 10) and TB mice (n = 10) per group. Survival curve graph represents at least three independent experiments. (B) Tumor weights of TB and TB-API mice on the last day of the study. (C) Body weights of CTRL, TB and TB-API mice on the last day of the study. (D) Spleen weights of CTRL, TB, and TB-API mice on the last day of the study. Graphs represented are the mean ± S.E.M of CTRL (n = 3), TB (n = 3) and TB-API (n = 3) mice of three independent experiments. *p<0.05 (by two-tailed Student’s *t* test).

API has been shown to have anti-tumor effects in a number of tumor models such as breast cancer and melanoma [40, 41]. To determine whether the effects of API on Ikaros *in vitro*, are also occurring *in vivo*, the impact of API treatment on survival and tumor burden was evaluated using TB mice. Treatment of TB mice with 25 mg/kg API caused an increase in their survival (Fig 2A) and a significant decrease in their tumor weight compared to vehicle-treated TB mice (Fig 2B). Next, we evaluated whether API treatment may have any toxicity effects *in vivo* by weighing and observing all mice three times a week for the duration of the study. Results showed that there was no significant difference in the weights of API treated compared to untreated TB mice at the end of the study (Fig 2C). A hallmark finding in TB mice is a pronounced splenomegaly, measured by a significant increase in spleen weights [42]. We found that *in vivo* API treatment reversed this PC induced splenomegaly and caused a significant reduction in spleen weights in TB-API compared to TB mice (Fig 2D).

### Apigenin partially stabilizes Ikaros expression, in vivo

**Fig 3:**
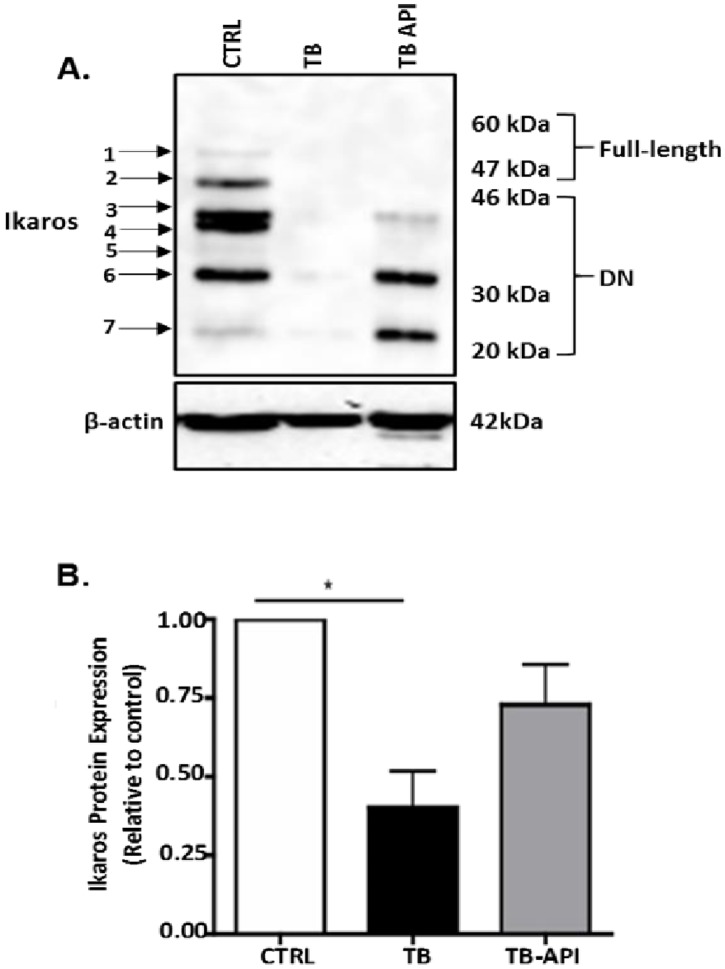
Apigenin partially stabilizes Ikaros expression, in vivo. (A**)** Western blot analysis of Ikaros protein expression in splenocytes from Control (CTRL), TB and TB-API mice. To control for equal protein loading, the blot was reprobed with an antibody specific to β-actin. The arrows on the left indicate observed Ikaros isoforms. **(**B) Representative quantification of normalized densitometric ratios of western blot data is shown. Graph represented is the mean ± S.E.M. of CTRL (n = 3), TB (n = 3) and TB-API (n = 3) mice of three independent experiments. *p<0.05, (by two-tailed Student’s *t* test).

Since our *in vitro* data shows that API can stabilize Ikaros expression, especially in the presence of murine Panc02 cells, we evaluated the effect of API treatment on Ikaros protein expression in an *in vivo* pancreatic tumor microenvironment. Western blot analyses revealed that API partially restored Ikaros expression in TB-API mice compared to TB mice (Fig 3A). More specifically, it appears that DN Ikaros isoforms, (described as less than 46 kDa) [43], were increased in TB-API compared to TB mice (Fig 3A).

### Apigenin inhibits CK2 activity and improves PP1 expression, in vivo

**Fig 4:**
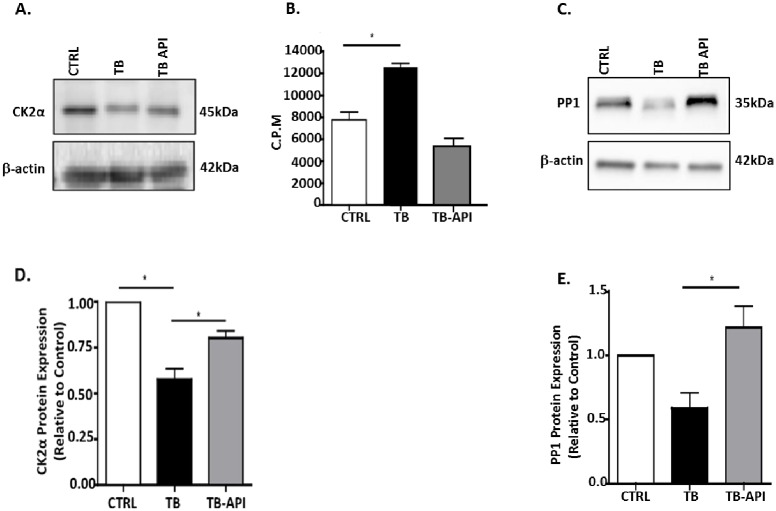
Apigenin inhibits CK2 activity and improves PP1 expression, in vivo. (A) Western blot analysis of CK2α protein expression in Control (CTRL), TB and TB-API splenocytes. (B) Counts per minute (C.P.M.) of CK2 activity in protein lysates from splenocytes from CTRL, TB and TB-API mice as assayed by an *in vitro* CK2 kinase assay (C) Western blot analysis of PP1 protein expression in CTRL, TB and TB-API splenocytes. (D) and (E) representative quantification of normalized densitometric ratios of western blots data of CK2 and PP1, respectively. To control for equal protein loading, the blots were reprobed with an antibody specific to β-actin. Graphs represented are the mean ± S.E.M. of CTRL (n = 3), TB (n = 3) and TB-API (n = 3) mice of three independent experiments. *p<0.05; (by two-tailed Student’s *t* test).

We previously published that key regulators of Ikaros expression CK2 (increased activity) and PP1 (down-regulated expression) were altered in TB mice [36]. Therefore we evaluated API’s effect on CK2 expression by western blot using an antibody specific to its catalytic alpha subunit. Splenocytes from TB-API mice showed a slight decrease in the molecular weight of CK2α compared to TB mice, similar to that seen in CTRL splenocytes (Fig 4A). Furthermore, we observed a significant increase in CK2α expression in TB-API compared to TB mice **(**Fig 4D)**.** To further delineate the effect of API on CK2 in our pancreatic TB model, we evaluated CK2’s activity and found that API treatment caused a reduction in CK2 activity in TB-API, compared to TB mice. However, this inhibition was not significant (Fig 4B). Next, we also evaluated PP1 expression (observed as doublets) in splenocytes found in CTRL mice, however the higher molecular weight isoform was absent in TB mice (Fig 4C**)**. In fact, the lower molecular weight PP1 isoform, which was present in splenocytes from CTRL, TB and TB-API mice was significantly increased in TB-API mice (Fig 4E**)**. These data strongly suggest that API is able to stabilize Ikaros expression *in vivo*, which may be mediated by its ability to inhibit CK2 activity and increase PP1 expression.

### Apigenin partially restores T cell homeostasis and immune responses, in vivo

**Fig 5:**
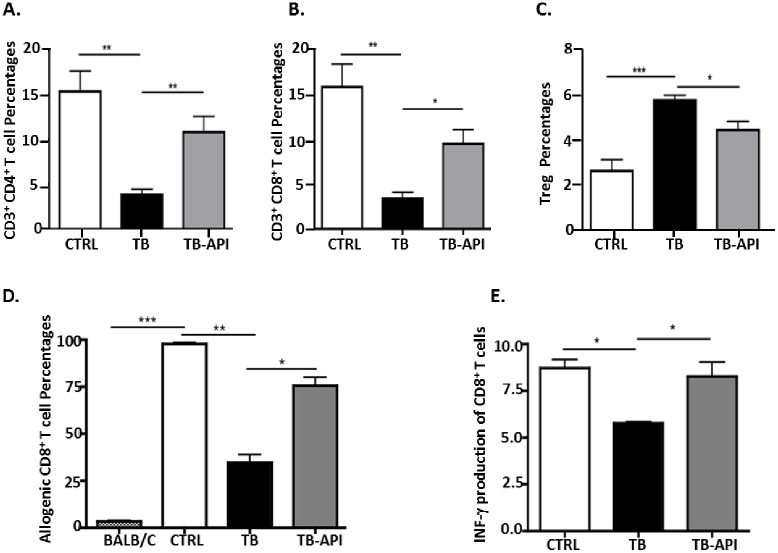
Apigenin partially restores T cell homeostasis and immune responses, in vivo. Flow cytometry analysis of (A) CD4^+^ T cells, (B) CD8^+^ T cells and (C) CD4^+^CD25^+^ Treg percentages in splenocytes from Control (CTRL), TB and TB-API mice. (D) Allogeneic CD8^+^ T cell proliferation responses of CFSE-labeled BALB/c splenocytes (responders) in response to CTRL, TB and TB-API splenocytes (stimulators) in a one-way mixed- leukocyte reaction (MLR), as analyzed by flow cytometry. (E) Flow cytometry analysis of IFN-γ production of CD8^+^ T cells in splenocytes from CTRL, TB and TB-API mice. Graphs represented are the mean ± S.E.M. of CTRL (n = 3), TB (n = 3) and TB-API (n = 3) mice of three independent experiments. *p<0.05; **p<0.005; ***p<0.0001(by two-tailed Student’s *t* test).

We previously published that T cell homeostasis was lost in TB mice [36]. To determine whether API-mediated rescue of Ikaros expression had any effect in the previously observed shift in T cell numbers in TB mice [36], we measured effector T cells (CD4^+^, CD8^+^ and Treg) percentages in TB-API and TB mice. Flow cytometry results show that splenocytes from TB mice had significant reduction in CD4^+^ (Fig 5A) and CD8^+^ T cell percentages (Fig 5B) but an increase in Treg percentages compared to CTRL mice (Fig 5C). However, flow cytometry results of splenocytes from TB-API mice had significantly increased CD4^+^ (Fig 5A) and CD8^+^ T cell percentages (Fig 5B) but reduced Treg percentages compared to TB mice (Fig 5C). These results suggest that Ikaros expression may in fact influence T cell development in TB murine model of PC. Next, we asked whether API-mediated an increase in effector T cell percentages and whether the reduction in Treg percentages could impact anti-tumor immune responses in TB mice. Therefore, we performed one-way allogeneic mixed leukocyte reaction (MLR) to address this question. In the MLR assay, splenocytes from CTRL, TB and TB-API mice were used as stimulators and co-incubated with CFSE-labeled BALB/c splenocytes, which were used as responders. As expected, TB whole splenocytes were deficient in their ability to prime allogeneic CD8^+^ T cell immune responses compared to CTRL splenocytes (Fig 5D). In contrast, TB-API whole splenocytes restored the ability to prime allogeneic responses compared to TB splenocytes (Fig 5D). Based on this observation, we hypothesized that API treated TB mice may have a higher rate of activated CD8^+^ T cells that produce IFN-γ which is critical to their effector function in eliminating tumor cells [44]. To address this question, we evaluated intracellular IFN-γ production of CD8^+^ T cells from splenocytes of CTRL, TB and TB-API using flow cytometry. Intracellular staining and flow cytometry analyses revealed that there were defects in CD8^+^ T cell IFN-γ production in TB mice compared to CTRL, which were significantly restored with API treatment (Fig 5E). These findings suggest a correlation between Ikaros expression, T cell development and immune responses in a pancreatic tumor microenvironment and ultimately points to a possible involvement of CK2 as a key regulator.

## Discussion

**Fig 6:**
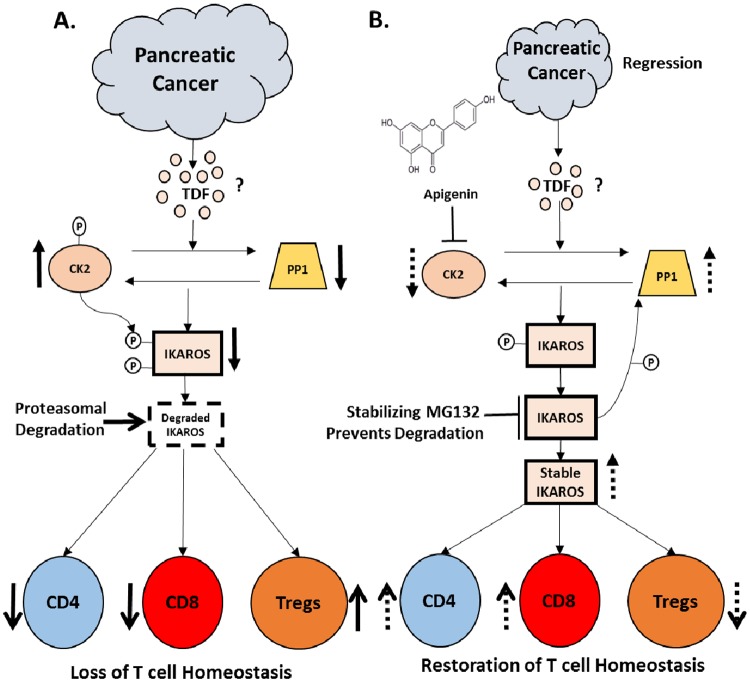
Proposed model. Ikaros’ regulation by CK2. (A) We propose that CK2 hyper-phosphorylates Ikaros, which facilitates its polyubiquitination and eventual protein degradation by the ubiquitin-proteasome system in PC. (B) We propose that API inhibits CK2 activity, stabilizing Ikaros expression by increasing PP1 activity and resulting in T cell homeostasis denoted by the broken arrows. We also propose that MG132 *in vitro* acts downstream by directly inhibiting that activity of the ubiquitin-proteasome system, preventing proteasomal degradation of Ikaros and thereby stabilizing its expression. Overall, our results suggest that *in vivo* API treatment increases T cell homeostasis and thus improves anti-tumor immune responses in PC microenvironment.

Recently, Song et al reported that inhibiting CK2 restored Ikaros tumor suppressor activity in clinical samples and pre-clinical xenograft models of leukemia [45]. Although widely studied in hematological malignancies [13], the role of Ikaros in solid cancers has not been fully investigated. We have previously identified the possible involvement of Ikaros in maintaining effector and regulatory T cell homeostasis in a pre-clinical PC model [36]. Our previous published data suggested that loss of Ikaros was a result of its ubiquitin-mediated proteasomal degradation in response to increased CK2 activity versus PP1 in a PC microenvironment (Fig 6A)[36]. In this current study, we make use of a selective CK2 inhibitor, apigenin (API), to further delineate the mechanism by which Ikaros is regulated and to provide functional evidence for its involvement in modulating T cell anti-tumor immune responses. *In vitro*, API stabilized Ikaros expression (IK1 and IK 2/3) in naïve splenocytes and prevented its downregulation in the presence of murine Panc02 cells, similar to MG132 treatment. *In vivo*, API treatment of TB mice improved survival, reduced tumor burden, reduced CK2 activity, increased PP1 expression and restored expression of some Ikaros isoforms (Fig 6B). In addition, API treatment of TB mice increased CD4/CD8^+^ effector T cell numbers while decreasing Treg numbers compared to TB mice (Fig 6B). Also, API treatment of TB mice restored the splenocytes’ ability to prime allogeneic CD8^+^ T cell responses in MLR. More importantly, API treatment of TB mice showed an increase in anti-tumor immune responses correlated with the increased production of intracellular IFN-γ from CD8^+^ T cells, *in vivo*. Our study sheds insight into Ikaros’ regulation of T cell immunity in PC and demonstrates evidence for a possible mechanism by which it is regulated. Regardless of the mechanism, the results of this study suggests that pharmacological CK2 inhibition restores Ikaros expression and can influence T cell immune responses in a murine PC model and other solid tumor models.

Phosphorylation of Ikaros by CK2 induces Ikaros degradation while dephosphorylation by PP1 maintains its stability [20, 21, 39]. *In vitro*, we found that API appeared to mimic the effects of MG132 by stabilizing Ikaros expression, causing the accumulation of its ubiquitination ladders. These data suggest that API may be similarly preventing ubiquitin-proteasomal degradation of Ikaros via its ability to inhibit CK2 activity. The combined effects of MG132 and API further provide evidence for this mechanism. As a result, our current working hypothesis is that API may be inhibiting the upstream effector of the pathway, CK2 and its ability to hyper-phosphorylate Ikaros further leading to its ubiquitination and degradation (Fig 6B). On the contrary, we propose that MG132 works downstream of this pathway by inhibiting the proteasome (Fig 6B). Ultimately, both inhibitors would lead to a more stable Ikaros expression. In addition, regarding our model Ikaros function is important for T cell homeostasis (Fig 6B).

Alternatively, API has also been reported to regulate proteasomal degradation. More specifically, API has been shown to potentially inhibit the chymotrypsin-like activity of the proteasome [40], similar to MG132 [46, 47]. It is therefore possible that API may stabilize Ikaros expression by inhibiting both CK2 and/or proteasomal activity, which needs to be further, investigated. Furthermore, clinically available proteasomal inhibitors exhibit some toxic effects [48], highlighting the need for safer alternatives such as natural, non-toxic compounds like API.

*In vivo*, API treatment improved survival and significantly reduced tumor weights of TB-API compared to TB mice. These findings suggest that API may have anti-tumor properties in murine PC. Although the frequency and dosage of API administered in our TB mice reduced CK2’s activity to half; this value however was not significant (p = 0.053). Therefore, in-depth pharmacokinetics and dose-dependent studies need to be done to determine a more effective dosage of API treatment for targeting CK2’s expression and/or activity in TB mice. Western blot analyses of CK2α showed an increase in its expression in splenocytes from TB-API compared to TB mice. However, CK2 expression in splenocytes of TB-API mice was accompanied by a reduction in MW, similar to that observed in splenocytes from CTRL mice. This suggests that API treatment may be inhibiting a posttranslational modification event of CK2. Phosphorylation of CK2 by kinases increases its activity [49, 50]. Therefore, this observation opens the intriguing possibility that API may reduce the activity of CK2 rather than the level of CK2. It will be crucial to evaluate other kinases such as ERK [49], and CDK1-cyclinB1 [51, 52], which may be responsible for modulating CK2’s activity in TB mice. Furthermore, western blot analyses from splenocytes showed a higher MW of PP1 isoform (demonstrated PP1 expression as doublets) found in CTRL and TB-API mice but absent in TB mice. PP1 protein expression was down-regulated but not significant (p = 0.0752) in TB mice compared to CTRL mice. API was able to significantly increase PP1 expression in TB mice with the restoration of the upper MW PP1 isoform (appearance of doublets) that was originally observed in CTRL mice. This data suggest that PC negatively impacts the phosphatase activity, which may be specific to only one of the several isoforms of PP1. Therefore it will be equally crucial to determine which PP1 isoform(s) activity is lost as a result of transcriptional or posttranslational modification events in PC microenvironments. In addition, it would be paramount to determine which PP1 isoform(s) responds to API treatment in TB (PC) mice.

API treatment appeared to increase the expression of Ikaros isoforms *in vivo*. Ikaros expression is critical for T cell immune balance. We previously published that full-length Ikaros isoforms (IK-1 and IK-2/3) in enriched CD3^+^ T cells were degraded in TB models [36]. Typically, the overexpression of DN isoforms is known to inhibit the activity of Ikaros and it is associated with T cell malignancies [53]. However, in this study we observed less degradation of Ikaros isoforms from splenocytes of TB-API compared to TB mice. Therefore, the identification of tumor-specific full-length and/or DN Ikaros isoforms and their functional impact on immune regulation and PC progression are warranted and are currently being investigated. We observed that API caused a decrease of CK2 activity in splenocytes from TB-API mice but there was no significant difference when compared to TB mice. However, it is possible that an increased dosage or more frequent treatments with API, may lead to a significant decrease in CK2 activity, which could lead to the up-regulation and the stability of more full-length Ikaros isoforms and consequently an increase in anti-tumor immune responses.

API treatment significantly increased CD4^+^ and CD8^+^ T cells but decreased Tregs percentages in TB mice. Our results showed functional evidence that API modulates immune responses in our TB mice since API treatment increased IFN-γ production of CD8^+^ T cells. This is an indication of restored CD8^+^ T cells’ activity and cytotoxic function [54, 55]. In addition, our data show that API also significantly increased the ability of antigen-presenting cells (APCs) to prime allogeneic CD8^+^ T cell immune responses. In a one-way MLR, allogeneic BALB/c CD8^+^ T cell responses were stimulated by APCs, from CTRL, TB and TB-API mice. Dendritic cells (DC) are the most potent APCs. Their function is often evaluated by their ability to induce proliferation of allogeneic T cells in MLR assays [56]. Therefore, the ability of TB-API splenocytes to effectively stimulate allogeneic CD8^+^ T cell proliferation may be a result of API’s effects on DC function, which has previously been reported [57]. API’s effects on DC function may be a result of its reduction of Treg percentages, which can inhibit DC function and T cell immune responses [8]. However, these Treg percentages were not fully restored to those of CTRL mice, which may also explain why allogeneic CD8^+^ T cell proliferation was not fully restored to CTRL levels. In addition, we have previously published that other immunosuppressive cells such as myeloid derived suppressor cells (MDSC) are expanded in TB mice [42]. MDSC are immature macrophages, dendritic cells and granulocytes [58]. API reduction in MDSC percentages may be a result of maturation or differentiation of these immature cells, thus producing mature DC, macrophages and other APCs, which could also account for the increased allogeneic immune responses. Our unpublished findings suggest that API reduces MDSC percentages, which may also account for the increased proliferation of allogeneic T cells from TB-API splenocytes in MLR assay (*unpublished* Ghansah et al., 2017). Overall, our results with API provide evidence that Ikaros may be specifically involved in regulating T cell immune responses in TB (PC) model.

In conclusion, this study highlights the importance of CK2 in regulating Ikaros expression and T cell immune responses in a solid pancreatic tumor microenvironment. Our results suggest that the natural flavonoid, API, may be therapeutically beneficial in stabilizing Ikaros expression via regulation of CK2 activity, thus restoring T cell homeostasis and enhancing anti-tumor immune responses in pancreatic cancer.

## References

[1] Oberstein PE, Olive KP. Pancreatic cancer: why is it so hard to treat? Therap Adv Gastroenterol. 2013;6(4):321–37. 10.1177/1756283X13478680 23814611 PMC3667471

[2] Morse MA, Hall JR, Plate JM. Countering tumor-induced immunosuppression during immunotherapy for pancreatic cancer. Expert Opin Biol Ther. 2009;9(3):331–9. 10.1517/14712590802715756 19216622

[3] Bazhin AV, Bayry J, Umansky V, Werner J, Karakhanova S. Overcoming immunosuppression as a new immunotherapeutic approach against pancreatic cancer. Oncoimmunology. 2013;2(9):e25736 10.4161/onci.25736 24327934 PMC3850017

[4] Kennedy R, Celis E. Multiple roles for CD4+ T cells in anti-tumor immune responses. Immunol Rev. 2008;222:129–44. 10.1111/j.1600-065X.2008.00616.x 18363998

[5] Kim HJ, Cantor H. CD4 T-cell subsets and tumor immunity: the helpful and the not-so-helpful. Cancer Immunol Res. 2014;2(2):91–8. Epub 2014/04/30. 10.1158/2326-6066.CIR-13-0216 24778273

[6] Xianjun Y, Shunrong J, Jin X, Wantong Y, Bin Q, Wenyan X, Bo Z, Yongfeng X. CD8+ T Cells Are Compromised In Human Pancreatic Cancer. Translational Medicine. p. 2161–1025.1000105.

[7] Fontenot JD, Gavin MA, Rudensky AY. Foxp3 programs the development and function of CD4+CD25+ regulatory T cells. Nat Immunol. 2003;4(4):330–6. Epub 2003/03/04. 10.1038/ni904 12612578

[8] Shevach EM. Biological functions of regulatory T cells. Adv Immunol. 2011;112:137–76. Epub 2011/11/29. 10.1016/B978-0-12-387827-4.00004-8 22118408

[9] Hiraoka N, Onozato K, Kosuge T, Hirohashi S. Prevalence of FOXP3+ regulatory T cells increases during the progression of pancreatic ductal adenocarcinoma and its premalignant lesions. Clin Cancer Res. 2006;12(18):5423–34. Epub 2006/09/27. 10.1158/1078-0432.CCR-06-0369 17000676

[10] Ghansah T, Vohra N, Kinney K, Weber A, Kodumudi K, Springett G, Sarnaik AA, Pilon-Thomas S. Dendritic cell immunotherapy combined with gemcitabine chemotherapy enhances survival in a murine model of pancreatic carcinoma. Cancer Immunol Immunother. 2013;62(6):1083–91. Epub 2013/04/23. 10.1007/s00262-013-1407-9 23604104 PMC3666559

[11] Yamamoto T, Yanagimoto H, Satoi S, Toyokawa H, Hirooka S, Yamaki S, Yui R, Yamao J, Kim S, Kwon AH. Circulating CD4+CD25+ regulatory T cells in patients with pancreatic cancer. Pancreas. 2012;41(3):409–15. 10.1097/MPA.0b013e3182373a66 22158072

[12] Rebollo A, Schmitt C. Ikaros, Aiolos and Helios: transcription regulators and lymphoid malignancies. Immunol Cell Biol. 2003;81(3):171–5. 10.1046/j.1440-1711.2003.01159.x 12752680

[13] John LB, Ward AC. The Ikaros gene family: transcriptional regulators of hematopoiesis and immunity. Mol Immunol. 2011;48(9–10):1272–8. 10.1016/j.molimm.2011.03.006 21477865

[14] Kathrein KL, Lorenz R, Innes AM, Griffiths E, Winandy S. Ikaros induces quiescence and T-cell differentiation in a leukemia cell line. Mol Cell Biol. 2005;25(5):1645–54. 10.1128/MCB.25.5.1645-1654.2005 15713624 PMC549358

[15] Urban JA, Brugmann W, Winandy S. Cutting Edge: Ikaros null thymocytes mature into the CD4 lineage with reduced TCR signal: A study using CD3{zeta} immunoreceptor tyrosine-based activation motif transgenic mice. J Immunol. 2009;182(7):3955–9. 10.4049/jimmunol.0802869 19299690 PMC2777666

[16] Winandy S, Wu P, Georgopoulos K. A dominant mutation in the Ikaros gene leads to rapid development of leukemia and lymphoma. Cell. 1995;83(2):289–99. 7585946 10.1016/0092-8674(95)90170-1

[17] Capece D, Zazzeroni F, Mancarelli MM, Verzella D, Fischietti M, Di Tommaso A, Maccarone R, Plebani S, Di Ianni M, Gulino A, Alesse E. A novel, non-canonical splice variant of the Ikaros gene is aberrantly expressed in B-cell lymphoproliferative disorders. PLoS One. 2013;8(7):e68080 10.1371/journal.pone.0068080 23874502 PMC3706598

[18] Georgopoulos K, Winandy S, Avitahl N. The role of the Ikaros gene in lymphocyte development and homeostasis. Annu Rev Immunol. 1997;15:155–76. 10.1146/annurev.immunol.15.1.155 9143685

[19] Gómez-del Arco P, Maki K, Georgopoulos K. Phosphorylation controls Ikaros's ability to negatively regulate the G(1)-S transition. Mol Cell Biol. 2004;24(7):2797–807. 10.1128/MCB.24.7.2797-2807.2004 15024069 PMC371126

[20] Popescu M, Gurel Z, Ronni T, Song C, Hung KY, Payne KJ, Dovat S. Ikaros stability and pericentromeric localization are regulated by protein phosphatase 1. The Journal of biological chemistry. 2009;284(20):13869–80. 10.1074/jbc.M900209200 19282287 PMC2679487

[21] Dovat S, Song C, Payne KJ, Li Z. Ikaros, CK2 kinase, and the road to leukemia. Molecular and cellular biochemistry. 2011;356(1–2):201–7. 10.1007/s11010-011-0964-5 21750978 PMC3665334

[22] Gurel Z, Ronni T, Ho S, Kuchar J, Payne KJ, Turk CW, Dovat S. Recruitment of ikaros to pericentromeric heterochromatin is regulated by phosphorylation. J Biol Chem. 2008;283(13):8291–300. 10.1074/jbc.M707906200 18223295 PMC2276389

[23] Wang H, Song C, Gurel Z, Song N, Ma J, Ouyang H, Lai L, Payne KJ, Dovat S. Protein phosphatase 1 (PP1) and Casein Kinase II (CK2) regulate Ikaros-mediated repression of TdT in thymocytes and T-cell leukemia. Pediatric blood & cancer. 2014;61(12):2230–5. 25214003 10.1002/pbc.25221 PMC4205270

[24] Ahmad KA, Wang G, Slaton J, Unger G, Ahmed K. Targeting CK2 for cancer therapy. Anticancer Drugs. 2005;16(10):1037–43. 16222144 10.1097/00001813-200511000-00001

[25] Pinna LA, Allende JE. Protein kinase CK2 in health and disease: Protein kinase CK2: an ugly duckling in the kinome pond. Cell Mol Life Sci. 2009;66(11–12):1795–9. 10.1007/s00018-009-9148-9 19387554 PMC11115792

[26] Litchfield DW. Protein kinase CK2: structure, regulation and role in cellular decisions of life and death. Biochem J. 2003;369(Pt 1):1–15. 10.1042/BJ20021469 12396231 PMC1223072

[27] Duncan JS, Litchfield DW. Too much of a good thing: the role of protein kinase CK2 in tumorigenesis and prospects for therapeutic inhibition of CK2. Biochim Biophys Acta. 2008;1784(1):33–47. 10.1016/j.bbapap.2007.08.017 17931986

[28] Seldin DC, Lou DY, Toselli P, Landesman-Bollag E, Dominguez I. Gene targeting of CK2 catalytic subunits. Mol Cell Biochem. 2008;316(1–2):141–7. 10.1007/s11010-008-9811-8 18594950 PMC3696998

[29] Landesman-Bollag E, Channavajhala PL, Cardiff RD, Seldin DC. p53 deficiency and misexpression of protein kinase CK2alpha collaborate in the development of thymic lymphomas in mice. Oncogene. 1998;16(23):2965–74. 10.1038/sj.onc.1201854 9662328

[30] Channavajhala P, Seldin DC. Functional interaction of protein kinase CK2 and c-Myc in lymphomagenesis. Oncogene. 2002;21(34):5280–8. 10.1038/sj.onc.1205640 12149649

[31] Patel D, Shukla S, Gupta S. Apigenin and cancer chemoprevention: progress, potential and promise (review). Int J Oncol. 2007;30(1):233–45. 17143534

[32] Mafuvadze B, Liang Y, Besch-Williford C, Zhang X, Hyder SM. Apigenin induces apoptosis and blocks growth of medroxyprogesterone acetate-dependent BT-474 xenograft tumors. Horm Cancer. 2012;3(4):160–71. 10.1007/s12672-012-0114-x 22569706 PMC10358033

[33] Ujiki MB, Ding XZ, Salabat MR, Bentrem DJ, Golkar L, Milam B, Talamonti MS, Bell RH, Iwamura T, Adrian TE. Apigenin inhibits pancreatic cancer cell proliferation through G2/M cell cycle arrest. Mol Cancer. 2006;5:76 10.1186/1476-4598-5-76 17196098 PMC1779363

[34] Strouch MJ, Milam BM, Melstrom LG, McGill JJ, Salabat MR, Ujiki MB, Ding XZ, Bentrem DJ. The flavonoid apigenin potentiates the growth inhibitory effects of gemcitabine and abrogates gemcitabine resistance in human pancreatic cancer cells. Pancreas. 2009;38(4):409–15. 10.1097/MPA.0b013e318193a074 19142175

[35] Hamacher R, Saur D, Fritsch R, Reichert M, Schmid RM, Schneider G. Casein kinase II inhibition induces apoptosis in pancreatic cancer cells. Oncol Rep. 2007;18(3):695–701. 17671722

[36] Nelson N, Xiang S, Zhang X, Gilvary D, Djeu J, Husain K, Malafa M, Vohra N, Pilon-Thomas S, Ghansah T. Murine pancreatic adenocarcinoma reduces Ikaros expression and disrupts T cell homeostasis. PloS one. 2015;10(1):e0115546 10.1371/journal.pone.0115546 25629611 PMC4309586

[37] Corbett TH, Roberts BJ, Leopold WR, Peckham JC, Wilkoff LJ, Griswold DP Jr., Schabel FM Jr. Induction and chemotherapeutic response of two transplantable ductal adenocarcinomas of the pancreas in C57BL/6 mice. Cancer research. 1984;44(2):717–26. 6692374

[38] Ghansah T, Paraiso KH, Highfill S, Desponts C, May S, McIntosh JK, Wang JW, Ninos J, Brayer J, Cheng F, Sotomayor E, Kerr WG. Expansion of myeloid suppressor cells in SHIP-deficient mice represses allogeneic T cell responses. Journal of immunology. 2004;173(12):7324–30. 10.4049/jimmunol.173.12.7324 15585856

[39] Song C, Li Z, Erbe AK, Savic A, Dovat S. Regulation of Ikaros function by casein kinase 2 and protein phosphatase 1. World journal of biological chemistry. 2011;2(6):126–31. 10.4331/wjbc.v2.i6.126 21765978 PMC3135859

[40] Chen D, Landis-Piwowar KR, Chen MS, Dou QP. Inhibition of proteasome activity by the dietary flavonoid apigenin is associated with growth inhibition in cultured breast cancer cells and xenografts. Breast Cancer Res. 2007;9(6):R80 10.1186/bcr1797 18300387 PMC2246179

[41] Caltagirone S, Rossi C, Poggi A, Ranelletti FO, Natali PG, Brunetti M, Aiello FB, Piantelli M. Flavonoids apigenin and quercetin inhibit melanoma growth and metastatic potential. Int J Cancer. 2000;87(4):595–600. 10918203 10.1002/1097-0215(20000815)87:4<595::aid-ijc21>3.0.co;2-5

[42] Pilon-Thomas S, Nelson N, Vohra N, Jerald M, Pendleton L, Szekeres K, Ghansah T. Murine pancreatic adenocarcinoma dampens SHIP-1 expression and alters MDSC homeostasis and function. PloS one. 2011;6(11):e27729 Epub 2011/12/02. 10.1371/journal.pone.0027729 22132131 PMC3222660

[43] Payne KJ, Huang G, Sahakian E, Zhu JY, Barteneva NS, Barsky LW, Payne MA, Crooks GM. Ikaros isoform x is selectively expressed in myeloid differentiation. J Immunol. 2003;170(6):3091–8. 12626565 10.4049/jimmunol.170.6.3091

[44] Qin Z, Schwartzkopff J, Pradera F, Kammertoens T, Seliger B, Pircher H, Blankenstein T. A critical requirement of interferon gamma-mediated angiostasis for tumor rejection by CD8+ T cells. Cancer Res. 2003;63(14):4095–100. Epub 2003/07/23. 12874012

[45] Song C, Gowda C, Pan X, Ding Y, Tong Y, Tan BH, Wang H, Muthusami S, Ge Z, Sachdev M, Amin SG, Desai D, Gowda K, Gowda R, Robertson GP, Schjerven H, Muschen M, Payne KJ, Dovat S. Targeting casein kinase II restores Ikaros tumor suppressor activity and demonstrates therapeutic efficacy in high-risk leukemia. Blood. 2015;126(15):1813–22. 10.1182/blood-2015-06-651505 26219304 PMC4600018

[46] Powers GL, Ellison-Zelski SJ, Casa AJ, Lee AV, Alarid ET. Proteasome inhibition represses ERalpha gene expression in ER+ cells: a new link between proteasome activity and estrogen signaling in breast cancer. Oncogene. 2010;29(10):1509–18. Epub 2009/12/01. 10.1038/onc.2009.434 19946334 PMC2837136

[47] Alexandrova A, Petrov L, Georgieva A, Kirkova M, Kukan M. Effects of proteasome inhibitor, MG132, on proteasome activity and oxidative status of rat liver. Cell Biochem Funct. 2008;26(3):392–8. Epub 2008/02/01. 10.1002/cbf.1459 18236383

[48] Adams J. The development of proteasome inhibitors as anticancer drugs. Cancer Cell. 2004;5(5):417–21. Epub 2004/05/18. 15144949 10.1016/s1535-6108(04)00120-5

[49] Ji H, Wang J, Nika H, Hawke D, Keezer S, Ge Q, Fang B, Fang X, Fang D, Litchfield DW, Aldape K, Lu Z. EGF-induced ERK activation promotes CK2-mediated disassociation of alpha-Catenin from beta-Catenin and transactivation of beta-Catenin. Mol Cell. 2009;36(4):547–59. Epub 2009/11/28. 10.1016/j.molcel.2009.09.034 19941816 PMC2784926

[50] Litchfield DW, Lozeman FJ, Cicirelli MF, Harrylock M, Ericsson LH, Piening CJ, Krebs EG. Phosphorylation of the beta subunit of casein kinase II in human A431 cells. Identification of the autophosphorylation site and a site phosphorylated by p34cdc2. J Biol Chem. 1991;266(30):20380–9. 1939094

[51] Litchfield DW, Lüscher B, Lozeman FJ, Eisenman RN, Krebs EG. Phosphorylation of casein kinase II by p34cdc2 in vitro and at mitosis. J Biol Chem. 1992;267(20):13943–51. 1629192

[52] Bosc DG, Slominski E, Sichler C, Litchfield DW. Phosphorylation of casein kinase II by p34cdc2. Identification of phosphorylation sites using phosphorylation site mutants in vitro. J Biol Chem. 1995;270(43):25872–8. 7592773 10.1074/jbc.270.43.25872

[53] Sun L, Crotty ML, Sensel M, Sather H, Navara C, Nachman J, Steinherz PG, Gaynon PS, Seibel N, Mao C, Vassilev A, Reaman GH, Uckun FM. Expression of dominant-negative Ikaros isoforms in T-cell acute lymphoblastic leukemia. Clin Cancer Res. 1999;5(8):2112–20. 10473095

[54] Hadrup S, Donia M, Thor Straten P. Effector CD4 and CD8 T cells and their role in the tumor microenvironment. Cancer Microenviron. 2013;6(2):123–33. 10.1007/s12307-012-0127-6 23242673 PMC3717059

[55] Ghanekar SA, Nomura LE, Suni MA, Picker LJ, Maecker HT, Maino VC. Gamma interferon expression in CD8(+) T cells is a marker for circulating cytotoxic T lymphocytes that recognize an HLA A2-restricted epitope of human cytomegalovirus phosphoprotein pp65. Clin Diagn Lab Immunol. 2001;8(3):628–31. Epub 2001/05/01. 10.1128/CDLI.8.3.628-631.2001 11329470 PMC96113

[56] Cao MD, Chen ZD, Xing Y. Gamma irradiation of human dendritic cells influences proliferation and cytokine profile of T cells in autologous mixed lymphocyte reaction. Cell Biol Int. 2004;28(3):223–8. Epub 2004/02/27. 10.1016/j.cellbi.2003.12.006 14984749

[57] Yoon MS, Lee JS, Choi BM, Jeong YI, Lee CM, Park JH, Moon Y, Sung SC, Lee SK, Chang YH, Chung HY, Park YM. Apigenin inhibits immunostimulatory function of dendritic cells: Implication of immunotherapeutic adjuvant. Mol Pharmacol. 2006;70(3):1033–44. 10.1124/mol.106.024547 16782805

[58] Nelson N, Szekeres K, Cooper D, Ghansah T. Preparation of myeloid derived suppressor cells (MDSC) from naive and pancreatic tumor-bearing mice using flow cytometry and automated magnetic activated cell sorting (AutoMACS). J Vis Exp. 2012(64):e3875 10.3791/3875 22733203 PMC3471281

